# Design and Interlaminar Stress Analysis of Composite Fan Blade Shank

**DOI:** 10.3390/polym17182445

**Published:** 2025-09-09

**Authors:** Yongjun Wu, Yukun Zhang, Zijian Wang, Lu Jin, Xu Tang, Xuyang Li, Yong Chen

**Affiliations:** 1 Xiangyang Hangtai Power Machinery Plant, Xiangyang 441002, China; yjw_ht@yeah.net; 2 School of Mechanical Engineering, Shanghai Jiao Tong University, Shanghai 200240, China; yukun-zhang@sjtu.edu.cn (Y.Z.); wangzijian.sjtu@sjtu.edu.cn (Z.W.); drjinl@sjtu.edu.cn (L.J.); t1996422@sjtu.edu.cn (X.T.); 3 School of Ocean and Civil Engineering, Shanghai Jiao Tong University, Shanghai 200240, China; lixuyang@sjtu.edu.cn; 4 Engineering Research Center of Gas Turbine and Civil Aero Engine, Ministry of Education, Shanghai 200240, China

**Keywords:** composite fan blade, shank, modeling design, ply design, interlaminar stress analysis

## Abstract

The fan blade shank serves as a critical transition structure connecting the airfoil and dovetail, with its geometric design significantly influencing the blade’s structural integrity. This study investigates the geometric configuration and static strength of the laminated composite fan blade shank, with emphasis on design methodology and analytical approaches. Utilizing Bézier spline curve techniques, two shank configurations—thickened and thinned—were developed for the laminated composite fan blade shank, followed by ply design and static strength analysis. The results demonstrate that high-stress regions in the laminated composite fan blade are predominantly located at the junction between the shank section and the leading edge of the dovetail. Furthermore, the thickened shank configuration effectively reduces the peak σ_33_ by approximately 15% and simultaneously alleviates the interlaminar shear stress σ_13_, without introducing adverse ply drop angles, which exhibits superior interlaminar stress resistance under tensile loading conditions.

## 1. Introduction

Carbon fiber composite materials exhibit exceptional specific modulus and strength characteristics. Composite fan blades are lighter than titanium alloy hollow fan blades and therefore contribute significantly to enhanced engine operating efficiency. Additionally, composite materials exhibit excellent vibration resistance, bird strike tolerance, and low noise characteristics. These attributes meet stringent airworthiness requirements, making composite fan blades integral to the lightweight design and efficient operation of aeroengines [1,2,3]. Currently, companies such as GE and CFM have successfully implemented the commercial application of composite fan blades [4]. Enhancing the bypass ratio of aeroengines and utilizing composite materials for lightweight design represent key development trends in commercial turbofan engines [3,4,5].

The fan blade consists of three main components: the airfoil, the dovetail, and the shank. The shank acts as the transition element connecting the airfoil and dovetail. The shank transfers loads from the airfoil to the dovetail. The geometric design of the shank plays a critical role in determining the overall structural strength of the blade. Surface modeling technology represents a fundamental aspect of computer-aided design and computer graphics. With the growing demand for surface design, modeling methods have evolved from early Bézier and B-spline curves to non-uniform rational B-spline curves (NURBS), which are widely applicable at present [6,7].

Significant research efforts have been devoted to the aerodynamic profiles and dovetail of fan blades in both domestic and international studies. The focus of research on curved geometries has predominantly been on the design optimization of aerodynamic blade surfaces [8]. Although parametric design methodologies for airfoils have reached considerable maturity, investigations into fan blade transition regions remain relatively scarce. Regarding the design of the dovetail, researchers have primarily concentrated on the factors that affect interlaminar strength. Bing Zhang et al. demonstrated through experimental studies and high-fidelity finite element simulations that the geometric discontinuity in the ply dovetail is the primary cause of delamination in skewed trapezoidal composite laminates. Additionally, they found that this phenomenon is highly sensitive to local defects [9]. Zhang Ting et al. further highlighted that the coupling of triangular gaps with ply termination significantly reduces the structural load-bearing capacity. To address this issue, they developed an automatic modeling tool to accurately predict the resulting strength reduction [10]. In severely skewed trapezoidal specimens that represent aerospace components, Stephen Hallett et al. successfully identified the delamination locations and ultimate loads induced by ply termination using high-fidelity models equipped with customized cohesive elements [11]. Khong Wui Gan et al. introduced a global–local delamination beam method, which can rapidly predict the initiation of delamination in multi-ply dovetail joints of thick-walled skewed trapezoidal composite plates [12]. Furthermore, Saeid Hosseinpour Dashatan et al. integrated in situ high-speed photography with explicit cohesive zone models to investigate how different ply configurations affect the dynamic delamination initiation, path, and stability in asymmetric GFRP skewed trapezoidal plates [13]. These studies offer valuable insights into the crack initiation mechanisms in complex ply configurations within transition sections. Zhang et al. established a static strength test platform for the dovetail of laminated composite fan blades and developed a corresponding finite element analysis model using the half-ply method. The experimental results aligned with the finite element predictions, confirming the validity of the finite element modeling approach [14].

At the same time, the fatigue and strength issues of blade connection structures, such as dovetail, have also received attention. Zeshuai Shen et al. investigated the crack initiation mechanisms of IN718 dovetail joints through room temperature and 650 °C high-cycle friction fatigue tests, combined with crystal plasticity models. They proposed the FFIP index to predict short crack propagation paths [15]. Hong Kyun Noh et al. developed a numerical model incorporating VUSDFLD/VUMAT subroutines, which validated the significant impact of thick–thin compression (TTC) effects and in-plane shear–tension coupling on delamination and ply fracture loads in a composite dovetail [16]. In the field of high-temperature reinforced materials, Yasuo Kogo et al. demonstrated through tensile testing and finite element analysis that the failure of three-dimensional reinforced C/C composite dovetail joints is controlled by average shear stress, and the peak shear stress at the shoulder is effectively mitigated during stress redistribution [17]. Yuhang Qin et al. explored the effects of size and ply orientation, revealing that slot stiffness dominates the tensile performance of carbon fiber dovetail joints, and appropriately increasing the 90° ply can reduce stress concentration [18].

Gao Pengfei et al. performed a photoelastic optimization design of turbine blade shank, reducing stress concentration and extending blade service life [19]. Wang Rui et al. proposed a cross-sectional line generation method for turbine blade shanks, enabling flexible surface parameter design based on control points and improving modeling efficiency [20]. Zhu Qian et al. summarized the surface characteristics and modeling requirements of turbine air-cooled blade shanks, introduced a longitudinal guide-line-based modeling method, and applied the minimum energy method for surface optimization [21]. Ren Yuan et al. developed a NURBS-based modeling method for fan blade shanks, predicted the resonance margin using a radial basis function network (RBFN), and optimized design parameters via the particle swarm optimization (PSO) algorithm, thereby enhancing the resonance margin of fan blades [22]. Chahine et al. utilized Bezier spline curves to generate the characteristic curve of the fan blade root section, ensuring a smooth connection between the blade airfoil and the dovetail [23].

Currently, publicly available literature primarily focuses on the design of the aerodynamic blade body and the dovetail, with limited attention given to the shaping and design analysis of the shank of fan blades. Unlike titanium alloy blades, changes in the geometric shape of composite blades require a redesign of the ply. Therefore, using the Bézier spline curve method, this paper designs the shank of a laminated composite fan blade, adjusts the ply sequence, conducts finite element static strength simulations, and analyzes the effect of shank geometry on the interlaminar stress of the fan blade.

## 2. Relevant Concepts and Definitions

### 2.1. Classical Laminate Theory

Composite fan blades are manufactured by stacking unidirectional fiber prepregs in various orientations, resulting in an irregular distribution of elastic properties through the thickness direction. An understanding of the theory about the behavior of laminated composites provides essential support for subsequent research and analysis.

Classical laminate theory is a fundamental theory widely applied in the mechanical analysis of laminate structures, based on the Kirchhoff assumption. This theory posits that laminates are elastic thin plates with no relative displacement between layers. The following assumptions underpin this theory [24]:

Kirchhoff assumption: Straight lines perpendicular to the mid-surface remain straight before and after deformation; strain in the thickness direction is neglected during deformation; After deformation, the straight line perpendicular to the midplane remains perpendicular to the midplane, and stress in the thickness direction can be neglected.

Interlaminar Deformation Assumption: The layers of the laminated plate are fully bonded, with consistent interlaminar deformation and no relative displacement.

Plane Stress Assumption: The layers of the laminated plate are in a plane stress state, with stress in the thickness direction being much smaller than in-plane stress and thus negligible.

#### 2.1.1. Strain–Displacement Relationship of Laminated Plates

Let the midplane of the laminated plate coincide with the xOy coordinate plane, where the x-axis direction is the fiber direction, the y-axis direction is the transverse direction perpendicular to the fibers, and the z-axis is perpendicular to the xOy plane. According to classical laminated plate theory, the displacements u_0_, v_0_, and w at any point in the laminated plate are

(1) $$\left\{\begin{array}{c} u_{0}=u_{0}\left(x,y\right)\\ v_{0}=v_{0}\left(x,y\right)\\ w=w\left(x,y\right) \end{array}\right.$$

According to the classic laminate theory assumption, the in-plane strain is

(2) $$\left\{\begin{array}{c} \varepsilon_{x}=\frac{\partial u}{\partial x}=\frac{\partial u_{0}}{\partial x}-z\frac{\partial^{2}w}{\partial x^{2}}\\ \varepsilon_{y}=\frac{\partial v}{\partial y}=\frac{\partial v_{0}}{\partial y}-z\frac{\partial^{2}w}{\partial y^{2}}\\ \gamma_{xy}=\frac{\partial u}{\partial y}+\frac{\partial v}{\partial x}=\frac{\partial u_{0}}{\partial y}+\frac{\partial v_{0}}{\partial x}-2z\frac{\partial^{2}w}{\partial x\partial y} \end{array}\right.$$

The strain of the middle plane can be subscribed as

(3) $$\left\{\begin{array}{c} \varepsilon_{x}^{0}=\frac{\partial u_{0}}{\partial x}\\ \varepsilon_{y}^{0}=\frac{\partial v_{0}}{\partial y}\\ \gamma_{xy}^{0}=\frac{\partial u_{0}}{\partial y}+\frac{\partial v_{0}}{\partial x} \end{array}\right.$$

The curvature of the middle plane can be represented as

(4) $$\left\{\begin{array}{c} \kappa_{x}=-\frac{\partial^{2}w}{\partial x^{2}}\\ \kappa_{y}=-\frac{\partial^{2}w}{\partial y^{2}}\\ \kappa_{xy}=-2\frac{\partial^{2}w}{\partial x\partial y} \end{array}\right.$$

Then the strain of any position in the laminate is

(5) $$\left[\begin{array}{c} \varepsilon_{x}\\ \varepsilon_{y}\\ \gamma_{xy} \end{array}\right]=\left[\begin{array}{c} \varepsilon_{x}^{0}\\ \varepsilon_{y}^{0}\\ \gamma_{xy} \end{array}\right]+z\left[\begin{array}{c} \kappa_{x}\\ \kappa_{y}\\ \kappa_{xy} \end{array}\right]$$

#### 2.1.2. Stress–Strain Relationship of Laminated Plates

The stress–strain relationship for the kth layer of laminate at a distance z from the middle plane is as follows:

(6) $${\left[\begin{array}{c} \sigma_{x}\\ \sigma_{y}\\ \tau_{xy} \end{array}\right]}_{k}={\left[\begin{array}{ccc} {\bar{Q}}_{11} & {\bar{Q}}_{12} & {\bar{Q}}_{16}\\ {\bar{Q}}_{12} & {\bar{Q}}_{22} & {\bar{Q}}_{16}\\ {\bar{Q}}_{16} & {\bar{Q}}_{26} & {\bar{Q}}_{66} \end{array}\right]}_{k}{\left[\begin{array}{c} \varepsilon_{x}\\ \varepsilon_{y}\\ \gamma_{xy} \end{array}\right]}_{k}$$

Among them, Q is the equivalent stiffness coefficient of each single layer.

Substituting Equation (5) into Equation (6), we obtain the stress–strain expression for laminated boards:

(7) $${\left[\begin{array}{c} \varepsilon_{x}\\ \varepsilon_{y}\\ \gamma_{xy} \end{array}\right]}_{k}={\left[\begin{array}{ccc} {\bar{Q}}_{11} & {\bar{Q}}_{12} & {\bar{Q}}_{16}\\ {\bar{Q}}_{12} & {\bar{Q}}_{22} & {\bar{Q}}_{16}\\ {\bar{Q}}_{16} & {\bar{Q}}_{26} & {\bar{Q}}_{66} \end{array}\right]}_{k}\left[\begin{array}{c} \varepsilon_{x}^{0}\\ \varepsilon_{y}^{0}\\ \gamma_{xy} \end{array}\right]+z{\left[\begin{array}{ccc} {\bar{Q}}_{11} & {\bar{Q}}_{12} & {\bar{Q}}_{16}\\ {\bar{Q}}_{12} & {\bar{Q}}_{22} & {\bar{Q}}_{16}\\ {\bar{Q}}_{16} & {\bar{Q}}_{26} & {\bar{Q}}_{66} \end{array}\right]}_{k}\left[\begin{array}{c} \kappa_{x}\\ \kappa_{y}\\ \kappa_{xy} \end{array}\right]$$

### 2.2. Bézier Curves

The dovetail of a composite fan blade features an airfoil-shaped curved surface on its upper side and a rectangular plane on the bottom side. The complex geometry of this structure demands precise mathematical description. Generating appropriate guiding curves is crucial for constructing a smoothly transitioning surface in the dovetail region.

The definition of a Bézier curve is as follows:

(8) $$p\left(t\right)=\sum_{j=0}^{n}a_{j}f_{j}\left(t\right),\;0\leq t\leq 1$$

Among them, $a_{j}$, (j = 0, 1, ⋯, *n*) is the coefficient vector, which is connected in order to form a Bezier polygon. $f_{j}(t)$, (j = 0, 1, ⋯, n) is the Bézier basis function, which is defined as:

(9) $$f_{j}\left(t\right)=\sum_{i=j}^{n}{\left(-1\right)}^{i+j}C_{n}^{i}C_{i-1}^{j-1}t^{i}$$

If l>k or l<0 and k≥0, then $C_{k}^{l}=0$; if l=0 and k≥0, then $C_{k}^{l}=1$. The Bernstein basis function defined by control vertex $b_{j}$ is:

(10) $$p\left(t\right)=\sum_{j=0}^{n}b_{j}B_{j,n}\left(t\right),0\leq t\leq 1$$

Among them, $b_{0}=a_{0}$, $b_{j}=b_{j-1}+a_{j}$, (j = 0, 1, ⋯, n) are control vertices; $B_{j,n}(t)$, (j = 0, 1, ⋯, n) are Bernstein basis functions, whose parameter expressions are:

(11) $$B_{j,n}\left(t\right)=C_{n}^{j}t^{j}{\left(1-t\right)}^{n-j}$$

Bézier curves have the following desirable properties, making this parameterization suitable for designing guide line shapes:

The start and end points of the Bézier curve correspond to the first and last vertices of the Bezier polygon, i.e., $p\left(0\right)=b_{0}$, $p\left(1\right)=b_{n}$. (b denotes control vertices, and p denotes curve nodes). The tangent directions at the start and end points of the Bezier curve align with the direction of the first and last edges of the characteristic polygon.

In the design of guide lines for the shank, the touch points are fixed and located at the top of the flow surface line and the dovetail, i.e., the upper and lower surfaces of the shank. The excellent properties of Bézier curves ensure that the guide lines maintain G0 continuity with the upper and lower surfaces of the root extension segment during adjustment, while also ensuring G1 continuity at the breakpoints.

## 3. Materials and Methods

**Figure 1:**
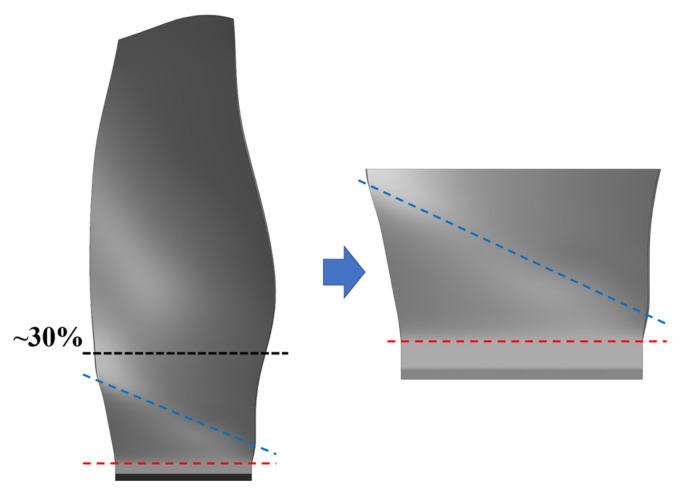
Schematic of the shank model, illustrating the retained section below 30% of the fan blade height, including the dovetail, shank, and a portion of the blade airfoil. The shank section is defined as the region between the blue and red lines.

The reference shank model in this study retains the section below 30% of the fan blade height. It includes the dovetail, shank, and a portion of the blade airfoil. An extension section is included in the dovetail to facilitate the ply design process. The shank model is shown in Figure 1. The shank section is defined as the region between the blue and red lines.

### 3.1. Materials

The unidirectional lamina properties and resin proxy used [14] in the finite element (FE) model are provided in Table 1 and Table 2.

### 3.2. Bézier Curves and Shank Design

The upper surface of the shank has an airfoil shape, while the lower surface is rectangular. Therefore, the shank demands a precise mathematical description of its geometry. Employing an appropriate method to generate guide lines is essential for constructing the shank’s transition surface. The shank geometry is designed by adjusting the control points of the guide lines. Variations in the shank’s configuration necessitate corresponding changes in ply design, leading to performance differences.

**Figure 2:**
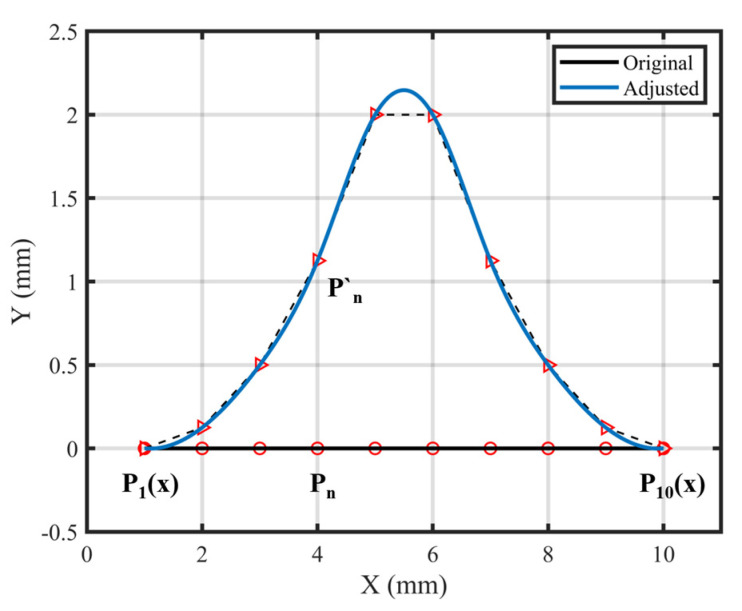
Representation of the control point on the guide line. Red circles are the control points on their original coordinate, while red triangles are the new location of the control points.

To minimize the impact of structural design on laminate design, the number of laminates on one side of the shank is restricted to a maximum of 10 layers. As illustrated in Figure 2, the maximum allowable displacement of the control point along the curve in space is constrained to within 10*d*, where *d* represents the thickness of a single prepreg layer.

**Figure 3:**
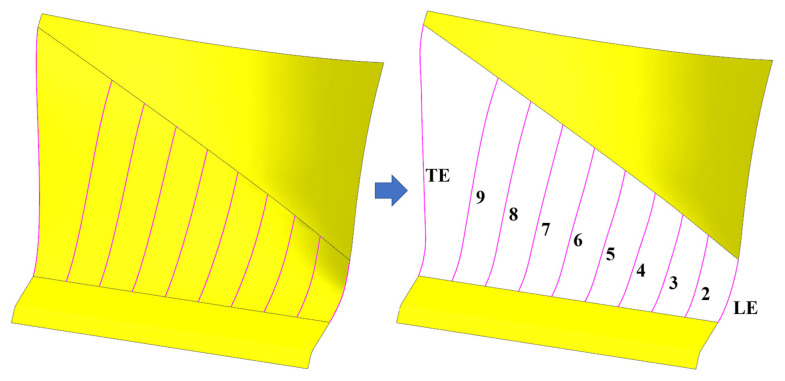
Guide lines on the shank surface, with the first (LE) and last (TE) guide lines positioned at the leading and trailing edges.

**Figure 4:**
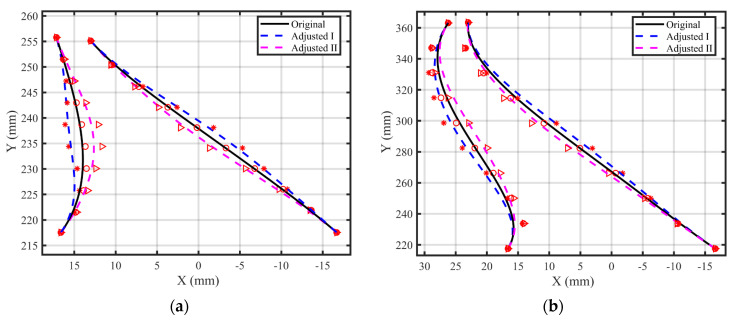
Diagram illustrating the adjustment of control points, showing the guide lines for the pressure surface on the left and the suction surface on the right, where (**a**) Line LE, (**b**) Line TE.

This study develops a design approach based on the existing shank model. The primary design steps are as follows: (1) Extract the guide lines of the pressure surface and suction surface. Ten guide lines are extracted, with the first and last positioned at the leading and trailing edges, as shown in Figure 3. (2) For each guide line, ten control points are extracted at equal arc length intervals. The first and last control points are fixed, while the remaining points are offset only in the ply direction, with a maximum offset of 2 mm, ensuring that the change in the number of plies on one side remains within ten layers. The displacement behavior of the control points follows Equation (12). New guide lines for the shank are generated using Bézier curves. Compared to the prototype design, Design I features a “convex” configuration, while Design II adopts a “concave” configuration. The maximum offset of the newly generated guide line is 2.15 mm, and the change in the number of plies on one side does not exceed eleven layers. (3) This process implements two shank designs: a thickened configuration (Design I) and a thinned configuration (Design II). The specific modeling results are shown in Figure 4. In each figure, the left side represents the guide line for the pressure surface, while the right side represents the guide line for the suction surface. (4) Following the completion of guide line modeling, the shank model is finalized using UG12 design software.

(12) $$\left\{\begin{array}{c} {P^{\prime }}_{n}\left(x\right)=2\times {\left(\frac{P_{n}\left(x\right)-P_{1}\left(x\right)}{L/2}\right)}^{2}\pm P_{n}\left(x\right),n=1,2,\cdots ,5\\ {P^{\prime }}_{n}\left(x\right)=2\times {\left(\frac{P_{n}\left(x\right)-P_{10}\left(x\right)}{L/2}\right)}^{2}\pm P_{n}\left(x\right),n=6,7,\cdots ,10 \end{array}\right.$$

where *L* is the arc length of the curve and *P_n_* (*x*) is the value at control point *n*.

### 3.3. Ply/Shuffle Design

**Figure 5:**
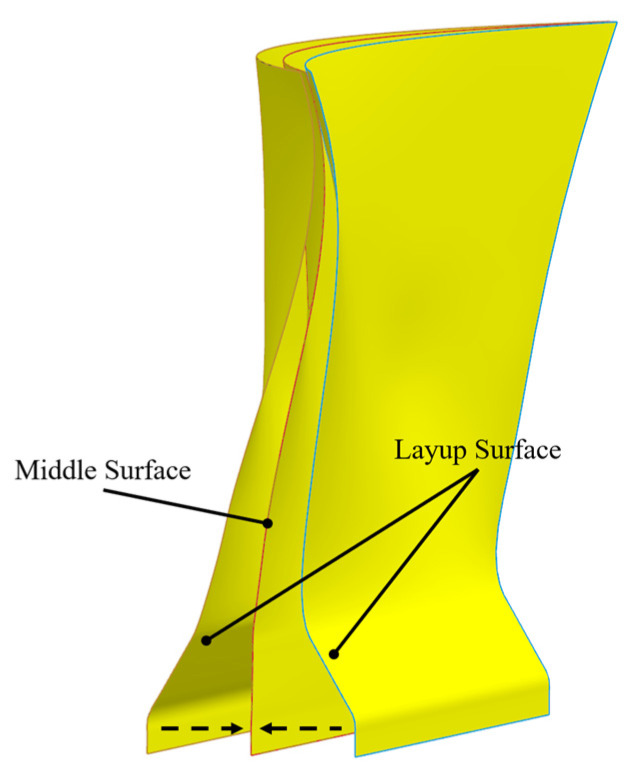
Ply model of the shank, illustrating the pressure side, suction side, and the middle surface used for composite ply generation.

As shown in Figure 5, the shank model has a thickness of 57.28 mm and consists of two main surfaces: the pressure side and the suction side, collectively referred to as layup surfaces. To facilitate plying, a middle surface is introduced between these two layup surfaces. Composite plies are generated between the layup surfaces and the middle surface to fill the shank.

**Figure 6:**
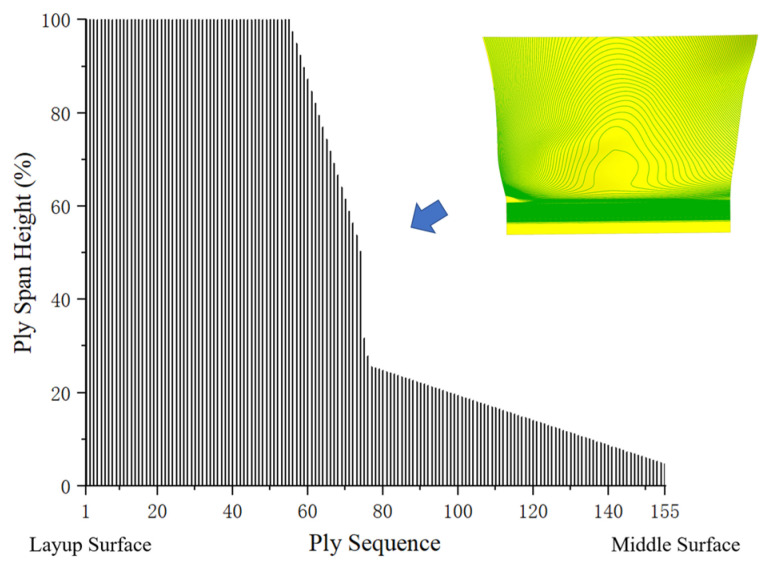
Ply height distribution before shuffling, showing the variation from the layup surface to the middle surface and the projection of ply layer contours on the middle surface.

The commercial software Fibersim17.0 is used to generate the ply layers. Each ply has a thickness of 0.185 mm [14], and the total number of plies in the shank is 155. The variation in ply height from the layup surface to the middle surface is shown in Figure 6, which also presents the projection of ply layer contours on the middle surface.

Short plies near the middle surface can create significant weak points in this area [25,26,27], failing to meet structural strength requirements, which makes ply shuffling essential. Important criteria for plying have been established and verified through analytical studies, numerical simulations, and engineering experience [28,29].

The plies are divided into several groups, defined as structural layers and insert layers. Based on ply height, the 1st to 94th layers are classified as structural layers, while the remaining layers are designated as insert layers. There are two types of structural layers: one with the stacking sequence [+45°/0°/−45°/0°], comprising 22 groups, and the other with the stacking sequence [+45°/0°/−45°/0°/+45°/0°], comprising 1 group. Similarly, for the insert layers, one stacking sequence is [0°/(+45°/0°/−45°/0°)2], comprising 4 groups, and the other is [0°/+45°/0°/−45°/0°], comprising 5 groups.

**Figure 7:**
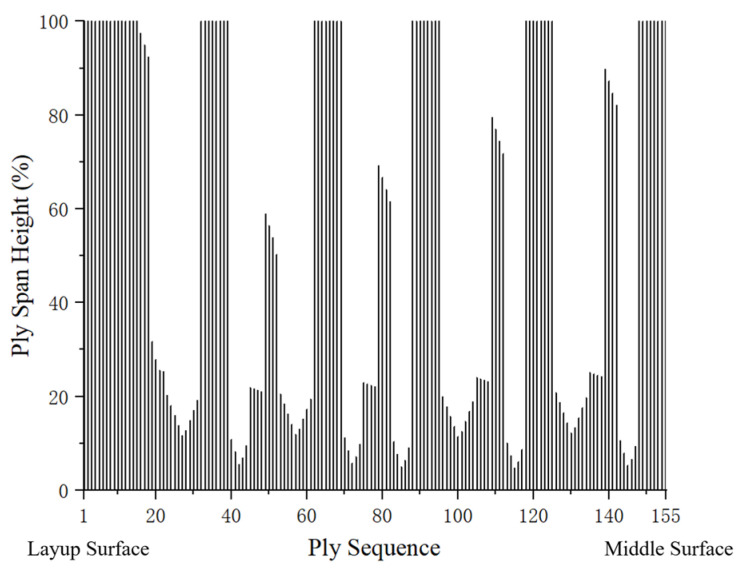
Ply height distribution after shuffling, demonstrating the adjustments made based on the established criteria.

Figure 7 illustrates the ply shuffling process based on the established criteria. To investigate the impact of different shank designs, two shapes of the laminated composite fan blade shank were developed: one thickened and the other thinned, with ply designs completed for each.

**Figure 8:**
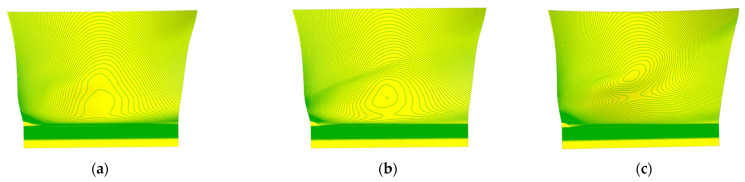
Projections of ply layer contours onto the middle surface, illustrating the inclusion of circular layers in the new designs, where (**a**) is original design, (**b**) is Design I, (**c**) is Design II.

Design I and Design II contain 156 and 161 plies, respectively, with their ply layer contours projected onto the middle surface, as shown in Figure 8. Compared to the original design, the new designs include circular layers. These circular layers, referred to as inner plies, are used to modify the thickness of the shank.

**Figure 9:**
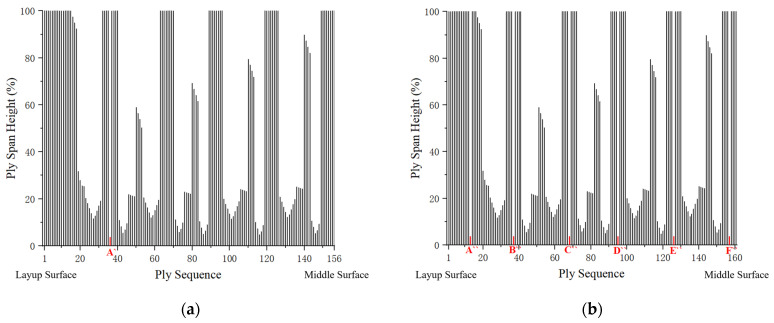
Positions of inner plies embedded between structural layers, indicated by the red labels, where (**a**) is Design I, (**b**) is Design II.

Layers with ±45° and 90° plies are more prone to failure. Considering the strength and compatibility of these layers, inner plies are set to 0°. As shown in Figure 9, inner plies are embedded at the locations indicated by the red labels. In this study, the embedded positions are located between two structural layers, which satisfy the criteria for plying.

### 3.4. Shank Structural Analysis

**Figure 10:**
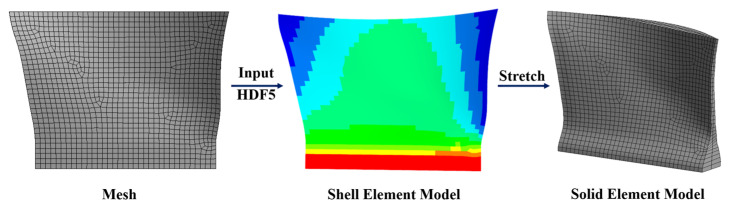
The procedure of building shank finite element model.

**Figure 11:**
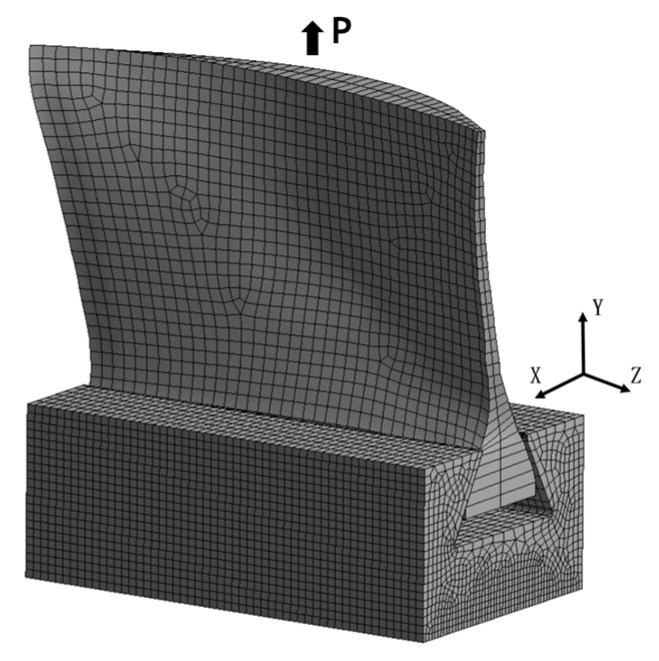
The finite element model of shank and disk.

The static strength analysis of the shank was performed using ANSYS ACP 2022R1. The primary objective is to examine the effect of different shank shape designs on the structural strength of the blade. Considering numerical analysis accuracy and time efficiency, a solid element, which is monolithic and contains half plies in the lay-up direction, is selected. Figure 10 illustrates the construction of the shank finite element model, while Figure 11 depicts the assembly of the shank and disk model. The parameters of the shank and disk models are provided in Table 3.

In this setup, the global x-axis is normal to the chord, the y-axis is aligned with the blade span, and the z-axis is along the chord. The disk is constrained at the bottom surface, where all displacements are set to zero in all directions. A tensile load of 38 MPa is applied to simulate the centrifugal force. The contact type is frictional, with a coefficient of friction set to 0.2.

## 4. Results and Discussion

Interlaminar delamination is a critical failure mode in laminated composite materials [30], and the influence of interlaminar stress on the structural strength of laminates is significant. This study analyzes interlaminar stress (shear stresses σ13 and σ23, and normal stress σ33) to evaluate the impact of different shank geometric designs on structural strength. Under tensile loading conditions, delamination primarily occurs in the mid-surface and layup surface regions of the laminated fan blade shank.

**Figure 12:**
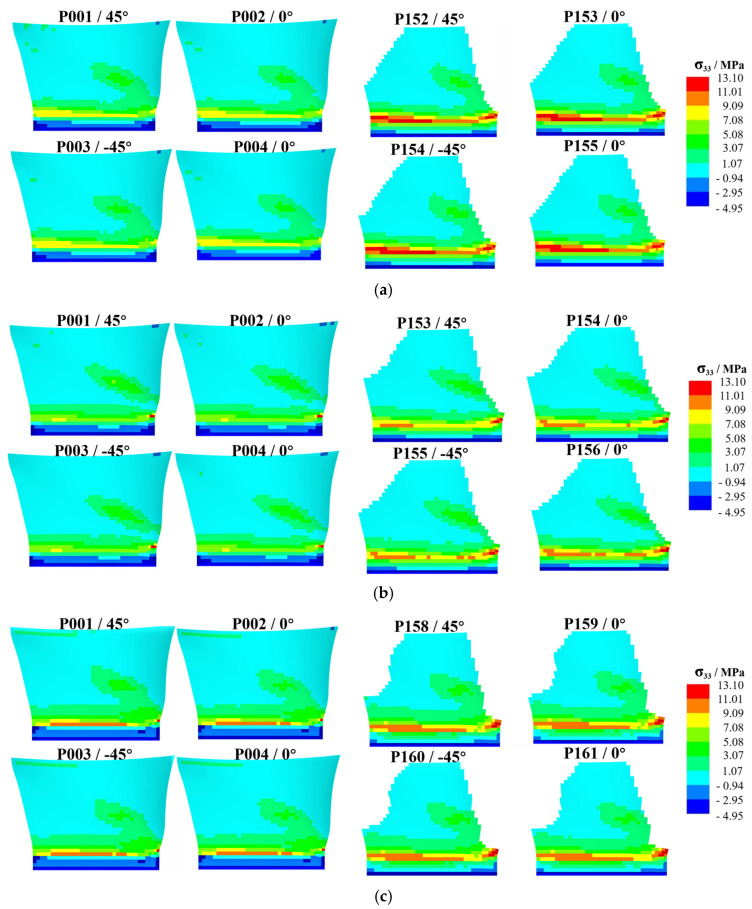
Contour plot of σ33 distribution, illustrating the high-stress areas on the middle and layup surfaces of the shank for different designs, where (**a**) is original design, (**b**) is Design I, (**c**) is Design II.

**Figure 13:**
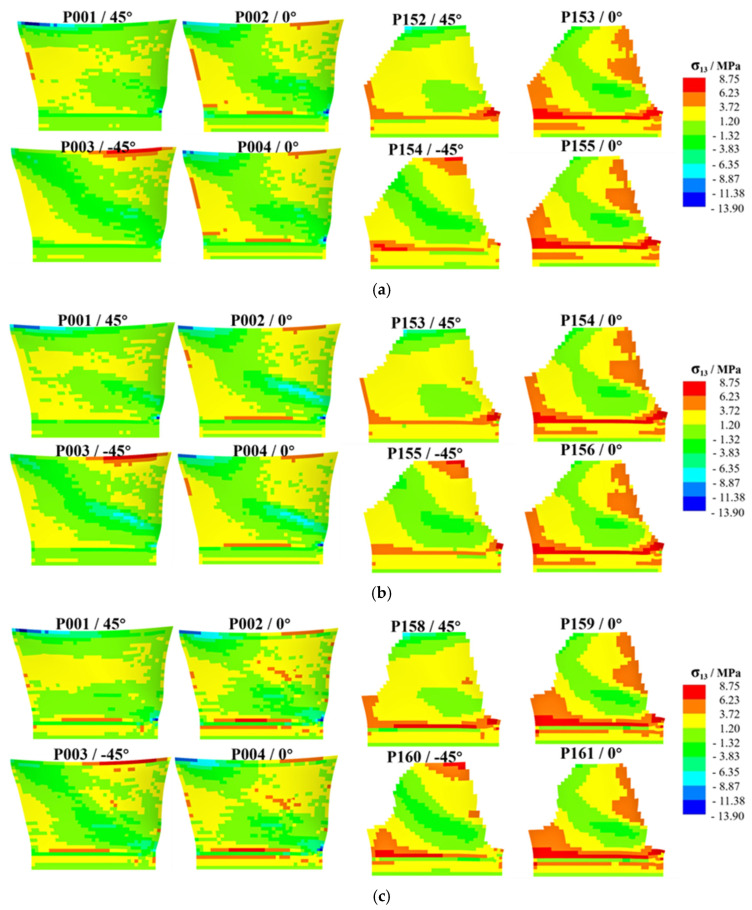
Contour plot of σ13 distribution, illustrating the high-stress areas on the middle and layup surfaces of the shank for different designs, where (**a**) is original design, (**b**) is Design I, (**c**) is Design II.

**Figure 14:**
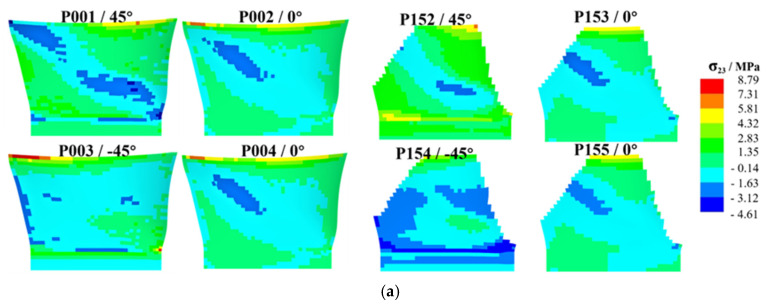

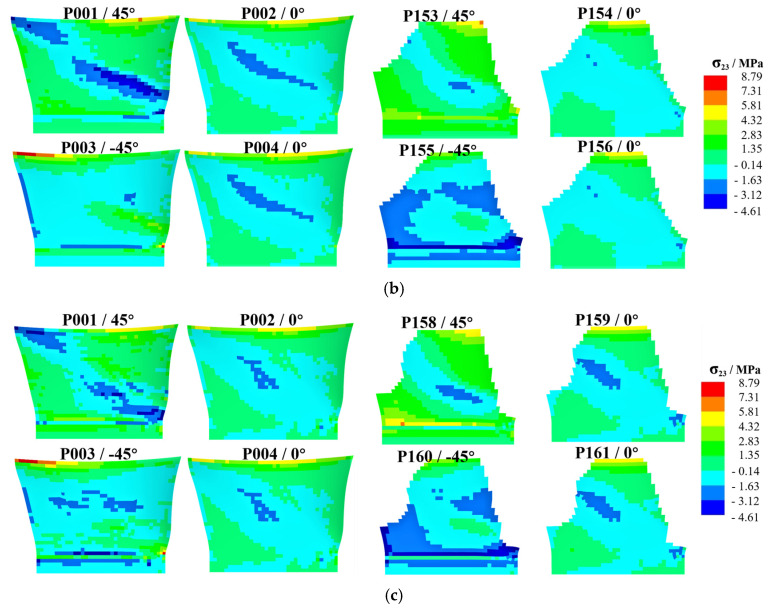
Contour plot of σ23 distribution, illustrating the high-stress areas on the middle and layup surfaces of the shank for different designs, where (**a**) is original design, (**b**) is Design I, (**c**) is Design II.

Taking the pressure surface side as an example, a group of laminates (four layers) is selected on the layup surface and mid-surface for analysis. As shown in Figure 12, Figure 13 and Figure 14, the high-stress area on the middle surface of the shank is extensive, with stress concentration occurring at the leading edge of the shank. The leading edge of the root section, due to its small thickness and high stress concentration, is more prone to delamination failure.

Figure 12 presents a comparison of σ33. Compared with the original design, the high-stress area on the middle surface of Design I is reduced, and the stress concentration at the leading edge is alleviated. Stress concentration is observed on the layup surface of Design I, but the stress value at the dovetail surface decreases. In Design II, the high-stress area on the middle surface is reduced, but the stress concentration at the leading edge worsens. Stress concentration is observed on the layup surface of Design II, and the stress value at the dovetail surface increases. Compared with Design I, the high-stress areas on the middle surface and layup surface of Design II are larger.

Figure 13 presents a comparison of σ13. Compared with the original design, the stress distribution on the mid-surface and layup surface of Design I shows no significant changes. The high-stress areas on the mid-surface and layup surface of Design II are increased. Figure 14 presents a comparison of σ23. Compared with the original design, the stress distribution of both Design I and Design II shows no significant changes.

**Figure 15:**
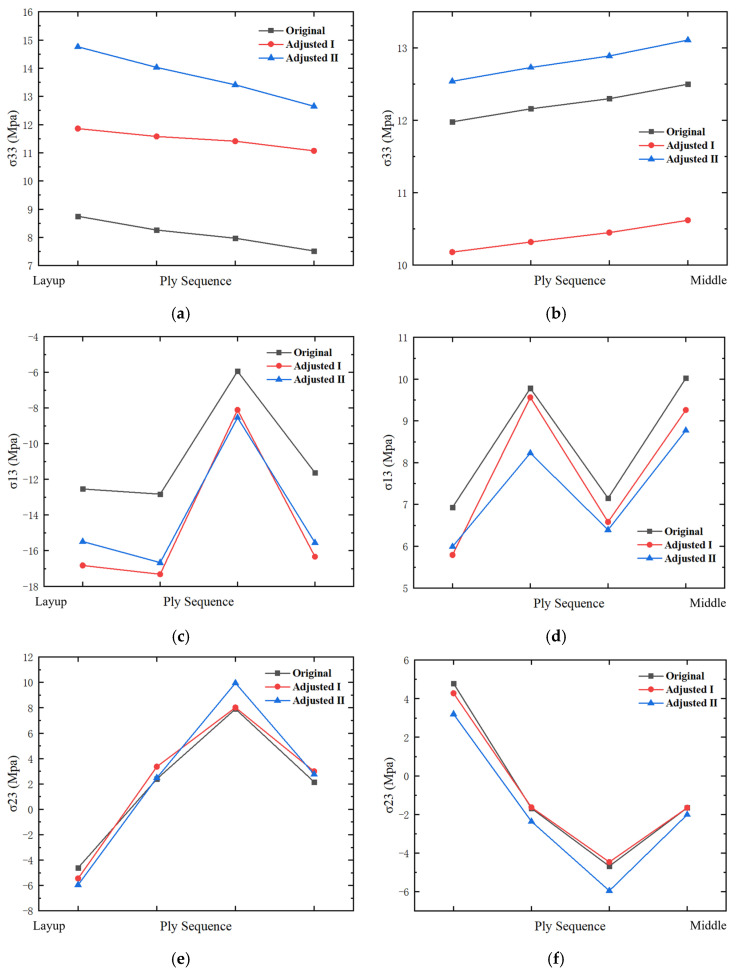
Interlaminar stress distribution across the ply sequence, highlighting stress trends at the leading edge of the shank for different designs, where (**a**) is σ33 on layup surface, (**b**) is σ33 on middle surface, (**c**) is σ13 on layup surface, (**d**) is σ13 on middle surface, (**e**) is σ23 on layup surface, (**f**) is σ23 on middle surface.

**Figure 16:**
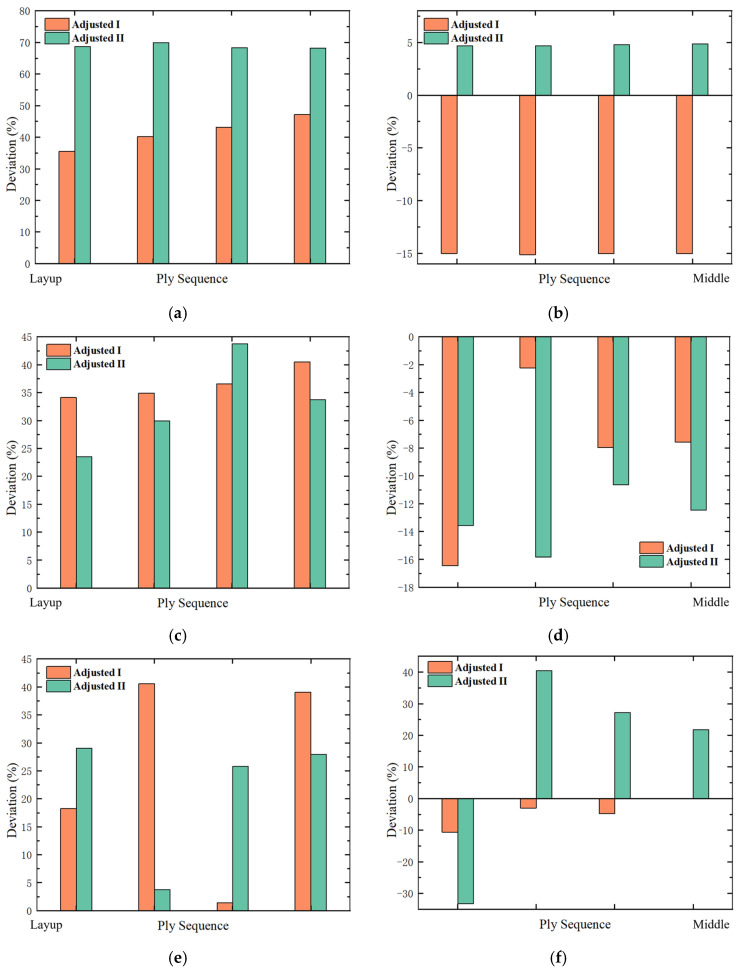
Comparison of maximum interlaminar stress deviations between the two design methods and the original design, where (**a**) is σ33 on layup surface, (**b**) is σ33 on middle surface, (**c**) is σ13 on layup surface, (**d**) is σ13 on middle surface, (**e**) is σ23 on layup surface, (**f**) is σ23 on middle surface.

Figure 15 provides a schematic diagram of the interlaminar stress distribution at the leading edge of the shank. The interlaminar stress distribution trends for all three designs are consistent. Figure 16 presents a bar graph comparing the maximum interlaminar stresses of the two design methods with the original design. Both designs result in an increase in σ33 on the layup surface. The σ33 in Design II increases by approximately 70%, while in Design I, it increases by less than 50%. On the middle surface, the σ33 in Design I decreases by approximately 15%, while in Design II, it increases by about 5%. The σ13 on the layup surface increases for both designs, while it decreases on the middle surface. The σ23 on the layup surface increases for both designs. The σ23 on the middle surface of Design I decreases.

Under tensile loading, the interlaminar stress of the first and last plies is relatively high, making them prone to delamination failure. Design I can reduce the stress concentration at the leading edge of the shank and the dovetail contact surface. In contrast, Design II exacerbates the situation. In summary, Design I helps reduce the interlaminar stress on the middle surface of the shank, providing valuable guidance for shank design.

## 5. Conclusions

In this study, the effect of stacking sequence control in the shank section of a composite fan blade was investigated, with a focus on the influence of local thickening on the distribution of interlaminar stresses. A detailed finite element model was developed, in which each ply was modelled using solid continuum elements. The interlaminar stresses σ_33_ and σ_13_ were computed and compared among three design configurations. The results revealed that the baseline design exhibited a relatively high interlaminar tensile stress (σ_33_) at the leading edge of the shank–dovetail transition, which may serve as a potential site for delamination initiation.

Design I (locally thickened shank) effectively reduced the peak σ_33_ by approximately 15% and simultaneously alleviated the interlaminar shear stress σ_13_, without introducing adverse ply drop angles. This improvement was achieved with only about a 1.8% increase in blade mass, demonstrating high stress-relief efficiency. In contrast, Design II (locally thinned shank) achieved a slightly better weight reduction (~1.2%) but led to increased stress concentration in critical areas, which may reduce structural reliability.

From an engineering perspective, local thickness tailoring provides a practical and manufacturable design strategy that can significantly improve the delamination resistance of composite blade structures. The resulting stress reduction implies higher overspeed capability and improved tolerance to in-service damage. This approach requires no changes to the material system or fabrication process and can be readily integrated into existing composite manufacturing workflows.

In summary, the thickened-shank design proposed in this work offers an effective and low-risk solution to enhance the durability and reliability of composite rotating components, such as fan blades. Future research may further consider material nonlinearity, interlaminar crack propagation, and thermal stress effects with the considerations of bird strike resistance and fatigue performance, and explore combined strategies such as high-fidelity FE modelling to comprehensively investigate interlaminar performance.

## Figures and Tables

**Table 1:** Unidirectional lamina properties used in the FE model.

Properties	Value
Density/(Kg/m^3^)	1490
Young’s Modulus in x Direction/GPa	121
Young’s Modulus in y&z Direction/GPa	8.6
Shear Modulus in xy&xz Direction/GPa	4.7
Shear Modulus in yz Direction/GPa	3.1
Tensile Stress Limit in x Direction/MPa	2231
Tensile Stress Limit in y&z Direction/MPa	29
Compressive Stress Limit in x Direction/MPa	−1082
Compressive Stress Limit in y&z Direction/MPa	−100
Shear Stress Limit in xy&xz Direction/MPa	60
Shear Stress Limit in yz Direction/MPa	32

**Table 2:** Resin properties used in the FE model.

Properties	Value
Density/(Kg/m^3^)	1160
Young’s Modulus/MPa	3780
Shear Modulus/MPa	1400
Poisson’s Ratio	0.35

**Table 3:** Shank and disk finite element model parameters.

Part	Element Sizes/mm	Nodes Number	Solid Number
Shank (Origin/I/II)	6	3960	2494
Disk	4	168,558	38,826

## Data Availability

The datasets generated and supporting the findings of this article are obtainable from the corresponding author upon request.
